# ISLR interacts with MGAT5 to promote the malignant progression of human gastric cancer AGS cells

**DOI:** 10.22038/IJBMS.2023.69372.15120

**Published:** 2023

**Authors:** Bin Zuo, Qiao Huang, Wei Yu, Jun Xu

**Affiliations:** 1 Department of Gastroenterology Surgery, Yichang Central People’s Hospital, The First College of Clinical Medical Science, China Three Gorges University, Yichang 443000, Hubei, China

**Keywords:** Gastric cancer, Invasion, ISLR, MGAT5, Migration, Proliferation

## Abstract

**Objective(s):**

Gastric cancer is a common malignant tumor with high morbidity and mortality. The present study aimed to investigate the role of the immunoglobulin superfamily containing leucine-rich repeat (ISLR) gene in gastric cancer and examine whether ISLR could interact with N-acetylglucosaminyltransferase V (MGAT5) to affect the malignant progression of gastric cancer.

**Materials and Methods:**

The expression of ISLR and MGAT5 in human normal gastric epithelial cells and human gastric cancer cells, and the transfection efficiency of ISLR interference plasmids and MGAT5 overexpression plasmids were all detected by reverse transcription-quantitative PCR (RT-qPCR) and western blot. The viability, proliferation, migration and invasion, and epithelial-mesenchymal transition (EMT) of gastric cancer cells after indicated transfection were detected by Cell counting kit-8 (CCK-8) assay, 5-ethynyl-2’-deoxyuridine (EdU) staining, wound healing assay, and transwell assay. The interaction between ISLR and MGAT5 was confirmed by co-immunoprecipitation. The expression of proteins related to migration, invasion, and EMT was detected by immunofluorescence and western blot.

**Results:**

As a result, ISLR was highly expressed in gastric cancer and was associated with poor prognosis. Interference with ISLR inhibited the viability, proliferation, migration, invasion, and EMT of gastric cancer cells. ISLR interacted with MGAT5 in gastric cancer cells. MGAT5 overexpression weakened the effects of ISLR knockdown on suppressing the viability, proliferation, migration, invasion, and EMT of gastric cancer cells.

**Conclusion:**

ISLR interacted with MGAT5 to promote the malignant progression of gastric cancer.

## Introduction

Gastric cancer is one of the common malignant tumors of the digestive tract. It is caused by a variety of causes, such as Helicobacter pylori infection, chronic atrophic gastritis, and various poor living habits (1). The morbidity and mortality of gastric cancer rank fifth and fourth among all kinds of malignant tumors all over the world (2). The five-year survival rate of early gastric cancer after radical surgery can reach 90%, but most gastric cancers in China are already in the advanced stage when diagnosed, and the five-year survival rate is low (3). The pathogenesis of gastric cancer is complex, and the specific mechanism of its occurrence and progress has not been clarified.

The Immunoglobulin superfamily containing the leucine-rich repeat (ISLR) gene (2.4-kb transcript) located on human chromosome 15q23-q24, is related to multiple genetic disorders (4). Research shows that ISLR is highly expressed in gastric cancer tissues, which is closely related to clinical prognosis. Gene ontology (GO) and Kyoto Encyclopedia of Genes and Genomes (KEGG) analysis have shown that the ISLR gene is involved in gastric cancer, the potential molecular mechanism of which may include DNA methylation, epithelial-mesenchymal transition (EMT), and immune cell infiltration (5). ISLR knockdown can promote apoptosis and suppress proliferation, migration, invasion, and glycolysis of non-small cell lung cancer (NSCLC) cells by inactivating the interleukin-6 (IL-6)/Janus kinase (JAK)/signal transducer and activator of transcription 3 (STAT3) pathway (6), and also inhibit the proliferation, migration, and invasion of colon cancer cells (7). Down-regulation of ISLR in stromal cells in mice obviously impairs intestinal regeneration and inhibits tumorigenesis in the colon (8). EMT refers to the transformation of epithelial cells into mesenchymal cells under specific physiological and pathological conditions, which is an important mechanism for tumor invasion and metastasis (9). The extracellular matrix (ECM) and EMT have been demonstrated to participate in the invasion and migration of gastric cancer (10-12). The ISLR gene has been reported to be involved in the activation of the EMT pathway (5). However, the specific role and mechanism of ISLR in gastric cancer have not been reported.

BioGRID database predicted a potential interaction between ISLR and N-acetylglucosaminyltransferase V (MGAT5). MGAT5 is an important N-glycan processing enzyme distributed in the Golgi apparatus, which can change the glycan structure of cell surface glycoproteins and thus aggravate the malignant transformation of cells and tumor metastasis (13, 14). Inhibition of MGAT5 activity can suppress the growth and metastasis of gastric cancer (15) and inhibit cell invasion and proliferation in glioblastoma (16). The increase in MGAT5 mRNA stability can promote stem-like cell phenotype in hepatocellular carcinoma (17). MGAT5 also promotes the proliferation and metastasis of breast cancer (18). 

Therefore, the present study aimed to investigate the role of the ISLR gene in gastric cancer and examine whether ISLR could interact with MGAT5 to affect the malignant progression of gastric cancer.

## Materials and Methods

***Cell culture***

Human normal gastric epithelial cells GES-1 and human gastric cancer cells (KE-39, MKN-45, and AGS) were provided by BioVector NTCC Inc. Cells were cultured in Roswell Park Memorial Institute (RPMI)-1640 medium supplemented with 10% fetal bovine serum (FBS), 100 μg/ml streptomycin, and 100 units/ml penicillin at 37 ^°^C with 5% CO_2_.

***Cell transfection***

Small interfering RNA (siRNA) of ISLR (si-ISLR) or siRNA negative control (si-NC) were synthesized by RIBOBIO (Guangzhou, China). MGAT5 overexpression vector pcDNA3.1-MGAT5 (Oe-MGAT5) and empty plasmid (Oe-NC) were purchased from MiaolingBio (Wuhan, China). The gastric cancer cells were seeded into six-well plates at the density of 2×10^5^ per well and cultured for 24 hr. Then, gastric cancer cells were transfected with the aforementioned plasmids using Lipofectamine 2000 (Invitrogen, Thermo Fisher Scientific) in a serum-free medium according to the manufacturer’s protocol for 48 hr prior to follow-up experiments.

***Reverse transcription-quantitative PCR (RT-qPCR)***

Total RNA was extracted from gastric cancer cells with Trizol (Invitrogen) and cDNA was synthesized with M-MLV (Vazyme, Nanjing, China) according to the instructions. RT-qPCR assay was performed using SYBR Green master mix (Vazyme) on an ABI Prism 7500 Sequence Detector (Applied Biosystems, USA). GAPDH was used as an internal reference. The relative expression of ISLR and MGAT5 in gastric cancer cells was calculated by the 2^−ΔΔCt^ method (19).

***Western blot***

The gastric cancer cells were lysed in radioimmunoprecipitation assay (RIPA) lysis buffer and the lysates were centrifugated at 12,000×g for 30 min at 4 ^°^C to obtain the proteins whose concentration was determined with the Bicinchoninic acid (BCA) method. The protein sample (50 µg) was separated by sodium dodecyl-sulfate polyacrylamide gel electrophoresis (SDS-PAGE) and then transferred to polyvinylidene fluoride (PVDF) membranes. After blocking with 5% non-fat dry milk in Tris-buffered saline with Tween-20, membranes were incubated with ISLR, MGAT5, matrix metalloproteinase-2 (MMP-2), matrix metalloproteinase-9 (MMP-9), E-cadherin, N-cadherin, Snail, and GAPDH at 4 ^°^C overnight. The membranes were incubated with horseradish peroxidaselabeled goat-anti-rabbit IgG antibody at room temperature for 1 hr the next day. Immunoreactive proteins were detected using enhanced chemiluminescence (ECL) Plus (Thermo Fisher Scientific), followed by analysis on a gel imaging analysis system (Gel Doc 1000; Bio-Rad).

***Cell counting kit-8 (CCK-8) assay***

The gastric cancer cells were seeded in a 96-well plate at the density of 800 cells per well and cultured in a 5% CO_2_ incubator at 37 ^°^C for 24 hr. Afterward, 10 μl CCK-8 (Beyotime) was also added to cells at 24, 48, and 72 hr, according to the instructions, and continuously incubated for 1 hr. The absorbance was measured at 450 nm with a Multiskan GO microplate reader (Thermo Scientific).

***5-Ethynyl-2’-deoxyuridine (EdU) staining***

EdU staining was conducted with an EdU kit (BeyoClick™ EdU Cell Proliferation Kit with Alexa Fluor 488, Beyotime). The gastric cancer cells (2×10^4^ cells/well) were seeded in 24-well plates and cultured for 72 hr. Next, cells were incubated with EdU for 2.5 hr, fixed with 4% paraformaldehyde for 20 min, and stained with Hoechst 33342 for 10 min. Finally, the stained cells were observed and photographed by an inverted fluorescence microscope.

***Immunofluorescence***

The gastric cancer cells were fixed in 4% paraformaldehyde for 10 min, washed with phosphate-buffered saline (PBS) for 5 min, and permeabilized with 0.1% Triton X-100 for 30 min. The slides were incubated with Ki-67 antibody overnight at 4 ^°^C, followed by incubation with a fluorescein isothiocyanate (FITC)-conjugated secondary antibody for 1 hr at room temperature. Finally, cells were stained with 4’,6-diamidino-2-phenylindole (DAPI) for 5 min and observed by an inverted fluorescence microscope.

***Wound healing assay***

The gastric cancer cells were seeded into six-well plates at the density of 2×10^5^ per well and cultured until the cells reached full confluence. The confluent cells were scratched by a sterilized 10-μl pipette tip. The detached cells were removed following PBS washing. After 24 hr, the migrated cells were observed by an inverted microscope, and the wound area was measured using the Image J software package.

***Transwell assay***

The gastric cancer cells were suspended in a serum-free medium at a density of 1×10^5^ cells/ml, and 100 μl cell suspension was seeded in the Matrigel-coated upper chambers. RPMI-1640 medium containing 10% FBS was added to lower chambers. After culturinng for 24 hr at 37 ^°^C with 5% CO_2_, the cells remaining in the upper chamber were removed and the cells that had invaded the bottom chamber were fixed with 95% ethanol, stained with 0.1% crystal violet for 10 min, and washed with PBS. The invaded cells were observed by an inverted microscope. 

***Co-immunoprecipitation***

Cell lysates were centrifugated at 12,000×g for 30 min at 4 ^°^C and then incubated with 30 μl protein A/G agarose beads (Roche) for 3 hr at 4 ^°^C to remove non-specific proteins. Equal amounts of protein (30 mg) were incubated with ISLR and MGAT5 antibodies overnight at 4 ^°^C. At last, precipitated proteins were incubated at 95 ^°^C for 5 min with Laemmli buffer and subjected to Western blot.

***Statistical analysis***

GraphPad Prism 8.0 (GraphPad Software, Inc.) was used for statistical analysis. All experimental data were shown as mean±standard deviation (SD) from at least three experiments. The statistical significance between the two groups was analyzed by Student’s t-test and among multiple groups using one-way analysis of variance (ANOVA) followed by Tukey’s *post hoc* test. *P*<0.05 was considered to be statistically significant.

## Results

ISLR is highly expressed in gastric cancer and is associated with poor prognosis.

**Figure 1:**
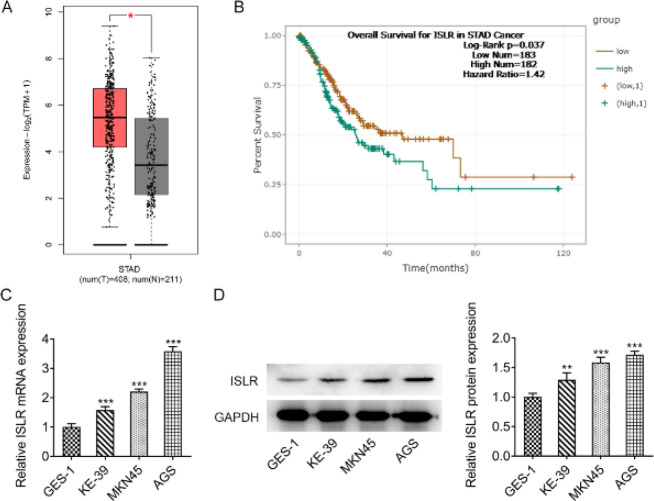
ISLR is highly expressed in gastric cancer and is associated with poor prognosis

GEPIA database indicated that ISLR was highly expressed in the tissues of gastric cancer patients (Figure 1A). Encyclopedia of RNA Interactomes (ENCORI) database showed that high expression of ISLR was significantly associated with low overall survival rate in gastric cancer patients (Figure 1B). The expression of ISLR was increased in gastric cancer cells compared with that in normal gastric epithelial cells, and ISLR expression was the highest in AGS cells. Therefore, AGS cells were selected for the subsequent experiments (Figure 1C and D).

***Interference with ISLR inhibits the proliferation of gastric cancer cells***

**Figure 2:**
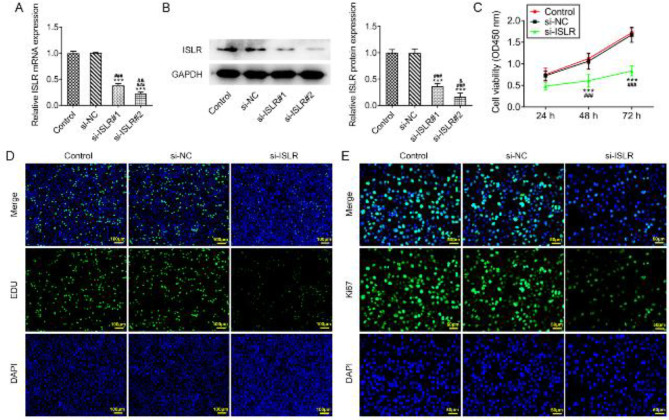
Interference with ISLR inhibits the proliferation of gastric cancer cells

When AGS cells were transfected with si-ISLR#1 and si-ISLR#2, the expression of ISLR was decreased and ISLR expression in si-ISLR#2 group was lower than that in the si-ISLR#1 group. Therefore, si-ISLR#2 was chosen for the following experiments (Figures 2A and 2B). The viability and proliferation of AGS cells were obviously suppressed by the knockdown of ISLR (Figures 2C and 2D). The expression of Ki-67 was correspondingly decreased in AGS cells transfected with si-ISLR (Figure 2E).

***Interference with ISLR inhibits the migration, invasion, and EMT of gastric cancer cells***

**Figure 3:**
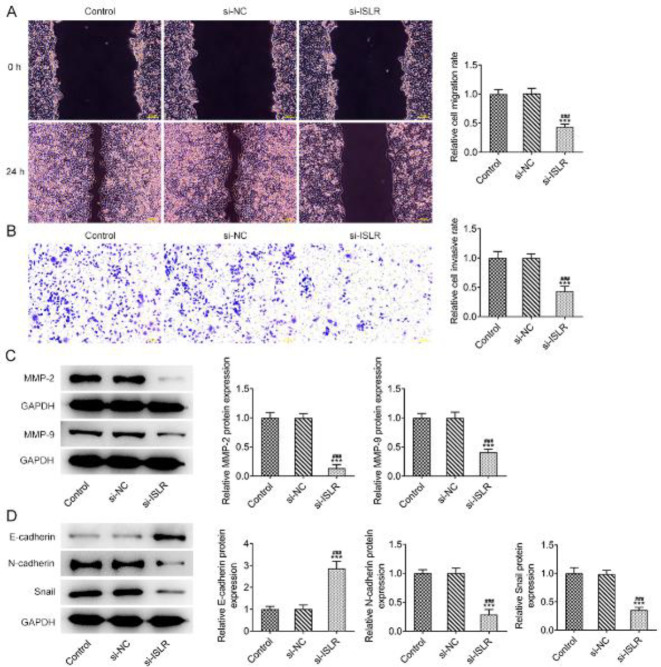
Interference with ISLR inhibits the migration, invasion, and EMT of gastric cancer cells

The migration and invasion of AGS cells were inhibited by the interference with ISLR (Figures 3A and 3B) with the declined expression of MMP-2 and MMP-9 (Figure 3C). Down-regulation of ISLR promoted the expression of E-cadherin while suppressing the expression of N-cadherin and Snail in AGS cells, indicating that EMT of AGS cells was inhibited (Figure 3D).

***ISLR interacts with MGAT5 in gastric cancer cells***

**Figure 4:**
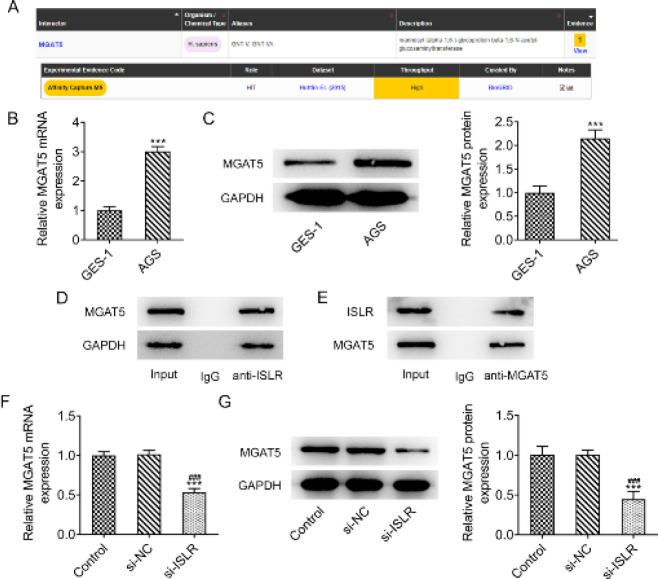
ISLR interacts with MGAT5 in gastric cancer cells

BioGRID database predicted a potential interaction between ISLR and MGAT5 (Figure 4A). The expression of MGAT5 in AGS cells was higher than that in GES-1 cells (Figures 4B and 4C). The results of co-immunoprecipitation indicated that ISLR interacted with MGAT5 (Figures 4D and 4E). The expression of MGAT5 in AGS cells was also inhibited by transfection of si-ISLR (Figures 4F and 4G).

***ISLR and MGAT5 synergistically promote the proliferation of gastric cancer cells***

**Figure 5:**
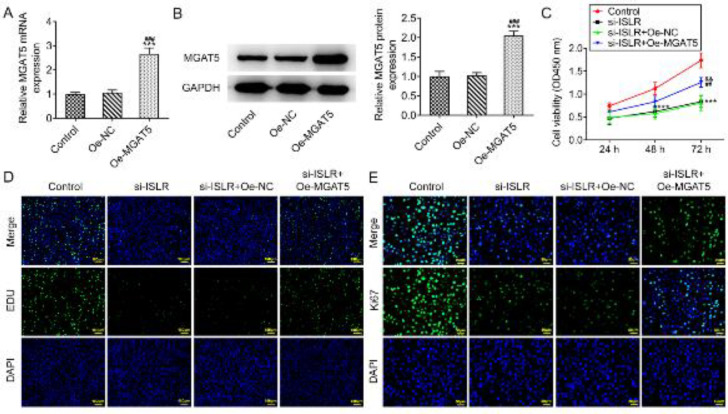
ISLR and MGAT5 synergistically promote the proliferation of gastric cancer cells

The expression of MGAT5 in AGS cells was up-regulated after transfection of Oe-MGAT5 (Figures 5A and 5B). Interference with ISLR suppressed the viability and proliferation of AGS cells and MGAT5 overexpression improved the viability and proliferation of AGS cells transfected with si-ISLR (Figures 5C and 5D). The expression of Ki-67 was down-regulated in the si-ISLR group, which was reversed by MGAT5 overexpression (Figure 5E).

***ISLR and MGAT5 synergistically promote the migration, invasion, and EMT of gastric cancer cells***

**Figure 6:**
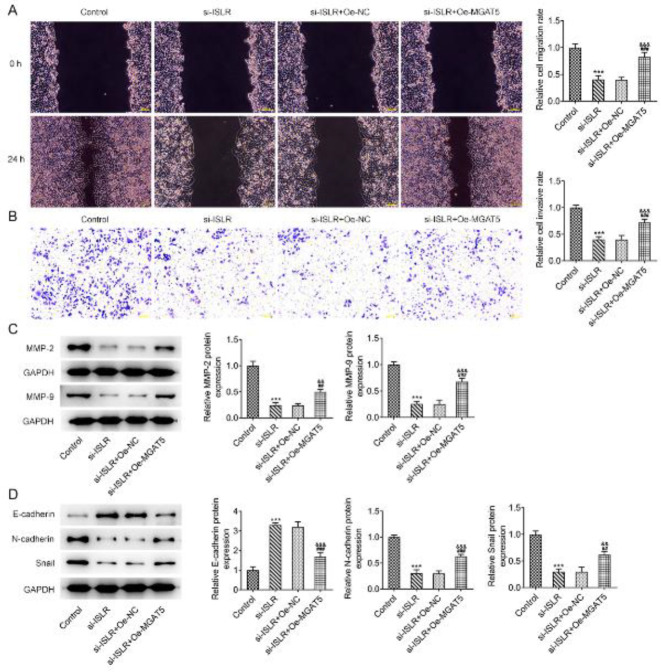
ISLR and MGAT5 synergistically promote the migration, invasion, and EMT of gastric cancer cells

The migration and invasion of AGS cells transfected with si-ISLR were inhibited and MGAT5 overexpression promoted the migration and invasion of AGS cells transfected with si-ISLR (Figures 6A and 6B). The expression of MMP-2 and MMP-9 in AGS cells transfected with si-ISLR was decreased, and MGAT5 overexpression increased the expression of MMP-2 and MMP-9 in AGS cells transfected with si-ISLR (Figure 6C). The expression of E-cadherin was increased while the expression of N-cadherin and Snail was decreased in AGS cells transfected with si-ISLR, which were reversed by the transfection of Oe-MGAT5.

(A) Prediction of ISLR expression in gastric cancer tissues by GEPIA database. (B) Correlation between ISLR high expression and overall survival rate of gastric cancer patients predicted by ENCORI database. (C and D) mRNA and protein expression of ISLR in normal gastric epithelial cells, and gastric cancer cells were detected by RT-qPCR and Western blot. **P<0.01 and ***P<0.001 vs GES-1 group

(A and B) Migration and invasion of AGS cells transfected with si-ISLR were analyzed by wound healing assay and transwell assay. (C and D) Expression of metastasis-related proteins and EMT-related proteins in AGS cells transfected with si-ISLR was analyzed by Western blot. *** *P<*0.001 vs Control group. ^###^ *P<*0.001 vs si-NC group

(A) BioGRID database predicted a potential interaction between ISLR and MGAT5. (B and C) mRNA and protein expression of MGAT5 in AGS cells and GES-1 cells was detected by RT-qPCR and western blot. ***P<0.001 vs GES-1 group. (D and E) Binding of ISLR and MGAT5 was confirmed by co-immunoprecipitation. (F and G) mRNA and protein expression of MGAT5 in AGS cells transfected with si-ISLR was detected by RT-qPCR and western blot. *** *P<*0.001 vs Control group. ^###^ *P<*0.001 vs si-NC group

(A and B) mRNA and protein expression of MGAT5 in AGS cells transfected with Oe-MGAT5 were detected by RT-qPCR and Western blot. *** *P<*0.001 vs Control group. ^###^ *P<*0.001 vs Oe-NC group. (C) viability of AGS cells transfected with si-ISLR and Oe-MGAT5 was detected by CCK-8 assay. *** *P<*0.001 vs Control group. ^##^ *P<*0.01 vs si-ISLR group. ^&&^ *P<*0.01 vs si-ISLR+Oe-NC group. (D) Proliferation of AGS cells transfected with si-ISLR and Oe-MGAT5 was observed by EdU staining. (E) Expression of Ki-67 in AGS cells transfected with si-ISLR and Oe-MGAT5 was detected by immunofluorescence

(A and B) Migration and invasion of AGS cells transfected with si-ISLR and Oe-MGAT5 were analyzed by wound healing assay and transwell assay. (C and D) Expression of metastasis-related proteins and EMT-related proteins in AGS cells transfected with si-ISLR and Oe-MGAT5 was analyzed by western blot. *** *P<*0.001 vs Control group. ^##^ *P<*0.01 and ^### ^*P<*0.001 vs si-ISLR group. ^&&^ *P<*0.01 and ^&&& ^*P<*0.001 vs si-ISLR+Oe-NC group

## Discussion

In recent years, due to the widespread popularization of endoscopy and the rapid development of early cancer diagnosis and treatment technology, the prognosis of patients with early gastric cancer has been improved to a certain extent (20). The high mortality rate of gastric cancer patients is mainly due to tumor recurrence and metastasis (21), which is a multistep pathophysiological process involving multiple gene modifications (22). Among them, abnormal gene expression is an important factor affecting the occurrence and development of gastric cancer (23). Previous studies by many scholars have found that multiple abnormally expressed genes are related to the molecular diagnosis or prognosis of gastric cancer (24-26), but few targeted drugs in gastric cancer have been applied clinically and achieved curative effects (27), and the improvement of prognosis and overall survival rate of patients is limited (28). The present study covered the effects of ISLR on gastric cancer cell proliferation, invasion, migration, and EMT to support the oncogenic role of ISLR in the malignant behaviors of gastric cancer cells, which could offer a potential biomarker for the diagnosis and treatment of gastric cancer.

EMT is characterized by the loss of epidermal cell phenotype and the acquisition of interstitial features, which play an important role in the process of tissue fibrosis, tumor invasion, and metastasis. In this process, tumor cells lose their polarity and intercellular connections and transform into motile stromal cells that can reach distant tissues through local infiltration or vasculature (29). E-cadherin is a protein responsible for intercellular adhesion between epithelial cells and maintenance of epithelial cell morphology, polarity, and integrity. Down-regulation of E-cadherin may weaken the adhesion between epithelial cells, which promotes the occurrence of EMT (30). In addition, the EMT process also secretes large amounts of MMP, which acts on the extracellular matrix, thus making tumor cells more likely to migrate, invade, and metastasize to distant areas (31). Knockdown of ISLR inhibits IL6induced proliferation, invasion, migration, EMT, and glycolysis in human NSCLC cells (6). Inhibition of the EMT signaling pathway can suppress the growth, migration, and invasion of colon cancer cells and weaken the pro-tumor properties of ISLR overexpression on colon cancer progression (7). The present study indicated that ISLR down-regulation could suppress the EMT process to inhibit the expression of MMP-2 and MMP-9, thereby suppressing the invasion and migration of AGS cells.

The alternation of tumor cell glycosylation is usually caused by the change of the expression of glycosyltransferase in the Golgi apparatus of tumor cells, the most common of which is the number and branch of N-linked glycans mediated by MGAT5. In the Golgi apparatus of cells, N-acetylglucosamine residues can be transferred to the glycan chain to form a multi-antennary structure (32). Knockdown of MGAT5 and up-regulation of Pcdhβ inhibits the growth of tumor cells, reduces the proportion of impregnated tumor cells, and reduces the formation of solid tumors (33). Wang *et al*. have found that MGAT5 reduces the protein level of RPTPkappa by adding β-1, 6-ethoylglucosaccharide to the N-sugar chain of the receptor protein tyrosinate phosphosinase kappa (RPTPkappa), thereby activating the epidermal growth factor receptor (EGFR) signaling pathway to promote cell migration (34). The published study has also reported that inhibition of MGAT5 activity impairs the growth and metastasis of gastric cancer (15). Here, AGS cells were transfected with si-ISLR and Oe-MGAT5 and the results showed that MGAT5 overexpression weakened the inhibitory role of si-ISLR in the aggressive behaviors of AGS cells, as evidenced by the finding that MGAT5 elevation promoted the viability, proliferation, invasion, migration, and EMT of AGS cells. 

## Conclusion

Taken together, the expression of ISLR and MGAT5 was increased in gastric cancer cells and interference with ISLR inhibited the proliferation, invasion, migration, and EMT of gastric cancer cells, which could be reversed by MGAT5 overexpression. This study is the first to demonstrate the suppressive role of ZBTB16 silencing in gastric cancer and present a novel regulatory relationship between ISLR and MGAT5 in gastric cancer. These factors could be used as molecular targets in the diagnosis and treatment of gastric cancer. Nevertheless, the present study has a limitation. In this study, we only discussed the regulatory effect of ISLR and MGAT5 on the progression of gastric cancer cells. Further *in vivo* experiments involved in transgenic animals will be performed in future investigations to support the conclusion obtained in this study.

## Authors’ Contributions

J X conceived and designed the current study. B Z, Q H, and W Y performed the experiments and data analysis. BZ wrote the manuscript, and J X revised the manuscript critically for important intellectual content. All authors have read and approved the final manuscript.

## Availability of Data and Material

The datasets used and/or analyzed during the current study are available from the corresponding author upon reasonable request.

## Conflicts of Interest

The authors declare that they have no competing interests.
